# Habitat Alterations by Viruses: Strategies by Tupanviruses and Others

**DOI:** 10.1264/jsme2.ME3302rh

**Published:** 2018-07-04

**Authors:** Hiroyuki Ogata

**Affiliations:** 1 Institute for Chemical Research, Kyoto University Gokasho, Uji, 611–0011 Japan

Viruses express phenotypes to alter their surrounding environments, thereby increasing their fitness. These surrounding environments include host metabolism and behavior. A recent study by Abrahão *et al.* (2) suggested a novel strategy for viral “sustainability” in which virions (*i.e.*, viral particles) modulate the population of their predator. The viruses they discovered were long tailed giant viruses named Tupanviruses. The genomes of Tupanviruses are 1.4–1.5 Mb of DNA, harboring the most complete set of genes for the translation apparatus among known viruses with up to 70 tRNAs and 20 aminoacyl-tRNA synthetases, implying the markedly reduced dependence of Tupanvirus replication on the host molecular machinery. Tupanviruses were consistently shown to infect a broad range of protist species including *Acanthamoeba* spp., *Vermamoeba vermiformis*, *Dyctiostelium discoideum*, and *Willartia magna*. This wide range of hosts is atypical among known giant viruses. Another interesting finding was that when infections by Tupanviruses were tested against the bacteria-feeder *Tetrahymena*, Tupanviruses failed to replicate in *Tetrahymena*; however, their virions exerted cytotoxic effects against this non-host protist. Therefore, Abrahão *et al.* performed *in vitro* experiments that simulated multiple virus-cell interactions, in which each of the Tupanviruses and Mimiviruses was cultured with their host (*Acanthamoeba*) and predator (*Tetrahymena*), the latter of which feed on giant viruses. The Tupanvirus population was maintained in the co-culture experiment because their virions diminished the activity of the predator (*Tetrahymena*) and successfully replicated in the host (*Acanthamoeba*). In contrast, Mimivirus particles, which are not toxic to *Tetrahymena*, were rapidly eaten by *Tetrahymena*, and, thus, did not successfully replicate. This finding indicates an ecological strategy not reported for viruses whereby their “seeds (8)” (*i.e.*, virions) are used as a weapon to control the population of their predators; however, this has not yet been validated in natural microbial communities.

Besides this outstanding discovery, a series of studies described the remarkable survival strategies of viruses that control viral host behavior and metabolism (33). Virophages are small viruses that require the dual hosts of a giant virus and eukaryotic cell for their replication (23). Metagenomic studies revealed that they are widespread in various environments (5, 30, 49, 51, 52). Fischer and Hackl (13) reported an interesting phenotype of mavirus, a virophage parasitizing the giant *Cafeteria roenbergensis virus* (CroV). It integrates into the genome of the marine protist *C. roenbergensis* and resides in the host genome as a provirophage. Genes of the mavirus provirophage are transcriptionally silent, but are activated when the host protist encounters the giant CroV virus, the predator of the protist. Activation of the provirophage leads to mavirus virion production, the unsuccessful replication of CroV, and lysis of the protist. Disseminated infectious mavirus virions then suppress CroV replication and, thus, enhance the survival of the host protist population. Therefore, the mavirus provirophage induces the altruistic behavior of its host, in which host cells choose death in order to protect their sisters. In this manner, mavirus increases the chances of replicating itself (through the activation by CroV infection and subsequent co-infection with CroV to the protist host) and maintaining the host population (by suppressing the propagation of giant CroV viruses).

Viral strategies to manipulate host behaviors are widely recognized in insect-virus systems (19, 47). A notable example is a baculovirus (*Lymantria dispar nucleopolyhedrovirus*; LdMNPV) that causes tree-top disease in infected lepidopteran larvae (18, 36). Infected larvae, or caterpillars, are more likely to die at elevated positions on the plant they feed on. This unusual behavior of infected larvae is considered to promote dissemination of the baculovirus from the top of the tree. In this case, the viral gene responsible for the modulation of host behavior has been identified. Hoover *et al.* (18) showed that the viral *egt* gene coding for an ecdysteroid UDP-glucosyl transferase, which affects the hormonal regulation of host development, is responsible for the climbing behavior (for death) of infected larvae.

Several viruses are known to be beneficial to their hosts (34). One example is an RNA virus (*Curvularia thermal tolerance virus*, CThTV) infecting a fungal endophyte (*Curvularia protuberate*), which is associated with a plant (the panic grass *Dichanthelium lanuginosum*). In this tripartite virus-fungus-plant symbiotic system, the RNA virus confers thermal tolerance on the plant, which grows on geothermal soil in Yellowstone National Park (25). This symbiotic interaction between viruses and their hosts, which may be more widespread in nature than is currently recognized (6, 31, 35), is a focus of an on-going Japanese scientific project, “Neovirology” (http://neo-virology.org/; (42)).

The viral modulation of host metabolism is also widely recognized, with host-derived metabolic genes often playing key roles in the control of host metabolism (20, 21, 29). A remarkable finding is the wide range of photosynthesis-related genes found in the genomes of viruses infecting cyanobacteria (24, 39, 48). Virally encoded photosynthesis-related genes (*e.g.*, *psbA* encoding the photosystem II core reaction center protein D1) are known to be expressed during infection and contribute to maintaining the photosynthetic activity of cyanobacterial cells during the lytic infection cycle of viruses (24). In this case, viruses, which eventually kill the infected hosts through lysis, help the infected cells to maintain energy production using the virally modulated photosynthesis machinery until viruses complete the production of virions that are released from the cells. It is important to note that 89% of cyanopodovirus genomes from sunlit oceans contain *psbA* (50), and 60% of environmental *psbA* are virally encoded (namely, only 40% encoded by photosynthetic cells) (38).

The above described phenotypes (or “extended phenotypes (11)”) of viruses do not make an exhaustive list of known viral alterations to their surrounding environments; however, even an exhaustive list only represents a very small proportion of the viral phenotypes in nature. There are 10^31^ viral particles on Earth (7), and previous studies estimated that ~20–40% of marine bacteria are killed daily by viruses (15, 40, 41). A recent study by single-cell genomics revealed that virally infected cells account for >60% of the cellular population in a hot spring microbial community (27). These findings indicate that an abundance of unicellular organisms are present not as “pure cells”, but as “virocells” (14), which refers to cells highjacked by viruses. The term “virocell” has been proposed to refer to the metabolically active stage of a virus (*i.e.*, a virocell=a virus). The estimated abundance of virocells among living cell populations in nature eloquently explains the ecophysiological impact of unseen viral phenotypes, such as the significant regulation of microbial food webs and biogeochemical cycles (10, 16, 17, 28).

In this issue of Microbes and Environments, Mihara *et al.* (26) reported that the taxonomic richness of a family of giant DNA viruses (*i.e.*, the “Megaviridae” family (4)) may exceed that of the domain Bacteria. The family “Megaviridae” includes the Tupanviruses and CroV cited above, and the number of isolated members together with other families of the order “Megavirales (9)” is currently increasing (1, 3, 12, 32, 43, 44). Only three domains of life have been identified for the cellular world, whereas 130 families have been described for the virosphere (see GenomeNet Taxonomy Summary; http://www.genome.jp/tools-bin/taxsummary). It currently remains unclear whether each of these viral families has the large genetic diversity of “Megaviridae”; however, notable diversity has been recognized in an RNA virus family (22), although its precise assessment will require an application of modern methods (*e.g.*, (37, 45, 46) to various environmental samples. Nevertheless, the diversity of viruses appears to represent the wide range of strategies for viral survival in environments. In other words, the diversity of viruses may represent the viral potential to change their surroundings, even possibly the global environment.
